# Recycling and Degradation Pathways of Synthetic Textile Fibers such as Polyamide and Elastane

**DOI:** 10.1002/gch2.202400163

**Published:** 2025-03-13

**Authors:** Pilar Chavez‐Linares, Sandrine Hoppe, Isabelle Chevalot

**Affiliations:** ^1^ Université de Lorraine CNRS LRGP Nancy F‐54000 France

**Keywords:** biotechnology, chemical recycling, elastane, polyamide, textile fibers, thermo‐mechanical recycling

## Abstract

Synthetic textile production is a major contributor to global waste growth, a phenomenon exacerbated by population growth and increased consumption. Global fiber production is expected to reach 147 million tons by 2030. New insights into recycling solutions are being developed. For example, progress has been made in recycling fibers such as polyester, including polyethylene terephthalate (PET), through the use of enzymes that can break specific bonds and return the material to its original state. However, this process must be carried out according to the nature of the polymer in question. In addition, the mixing of different synthetic fibers and the use of dyes make it difficult to develop a complete recycling process that separates the fibers and returns them to their original raw material. This review focuses on two types of fibers widely used in the textile industry, Nylon or polyamide (PA) and elastane (Spandex or Lycra), and explores the challenges and opportunities associated with their recycling.

## Graphical abstract

This review concerns two types of fibers widely used in the textile industry, polyamide and elastane, presenting common recycling types (mechanical and chemical) and focusing on biological pathways. Microbial degradation makes it possible to break down the material into carbon dioxide, water, methane, and mineral salts, while recycling by enzymatic processes should make it possible to return to the initial monomers constituting these polymers.

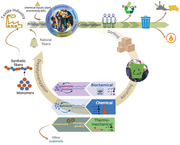

## 1 Introduction

Climate change is a major issue today and many industries are aware of their environmental impact. One of these industries is the textile industry, which is recognized as the third most polluting industry in the world.^[^ ^1^ ^]^ It is responsible for ≈10% of global carbon emissions and is a major contributor to water pollution, with dyeing and finishing processes accounting for ≈20% of clean water pollution. The rise of fast fashion has exacerbated these environmental problems, leading to increased waste and resource consumption, including the use of 2700 liters of water to produce a single cotton T‐shirt.^[^ ^2^ ^]^

In recent years, the phenomenon of fast fashion has become increasingly widespread. The growth of the world's population has led to an increase in clothing production. As a result, consumers tend to replace their clothes regularly in search of competitive prices. The majority of consumers now use synthetic fibers, which are derived from fossil fuels. Unlike natural fibers, which are derived from plants and animals, they are generally not biodegradable. This includes common synthetic fibers such as polyamide (PA), poly(ethylene terephthalate) – PET, acrylic, and Spandex, which resist biological degradation and pose significant environmental challenges.^[^ ^3^ ^]^ The large amount of clothing made with synthetic fibers creates a significant environmental problem due to the release of microplastics.^[^ ^4^ ^]^ Global fiber production is expected to increase from ≈116 million tons in 2022 to 147 million tons in 2030.^[^ ^5^ ^]^ Polyamide, or Nylon, is the second most widely produced synthetic fiber, accounting for ≈5% of global production at ≈4 million tons.^[^ ^6^ ^]^ In addition, polyamide has a high environmental impact,^[^ ^4^ ^]^ as its production can generate greenhouse gases such as nitrous oxide (N_2_O) and carbon dioxide (CO_2_), contributing to global warming.^[^ ^7^ ^]^ Another commonly used synthetic fiber is elastane (known as Spandex or Lycra), which is a polyurethane elastomer based on flexible poly(ethylene glycol) blocks and rigid polyurea blocks, usually elastane is blended with polyester, polyamide, and natural fibers to make stretch fabrics.^[^ ^8^ ^]^ This material is elastic and is often used for comfort and fit in casual wear, elastic corsetry fabrics, and hosiery.^[^ ^9^ ^]^ Elastane is often found in a large number of products in blended materials, which has a small volume production of ≈1.1% of global fiber production.^[^ ^6^ ^]^ Global elastane fiber production has been estimated at 1.2 million tons in 2022.^[^ ^5^ ^]^

Fiber blends are a major drawback for a recycling process in the textile industry. For example, the most common fiber blends are cotton with polyester and elastane with polyamide and with polyester.^[^ ^10^ ^]^ Thus, the separation of blended synthetic fibers is currently a major challenge for waste management. In addition, the presence of additives and dyes hinders the development of a recycling process toward a circular economy.^[^ ^11^ ^]^

According to research by T. Uekert and co‐workers,^[^ ^12^ ^]^ mechanical recycling of PET typically yields between 70% and 90%, depending on the quality of the input material and the efficiency of the recycling process. Chemical recycling processes, such as depolymerization, can achieve yields of ≈90% or higher, depending on the specific technology and conditions used. These processes can effectively break down PET into its monomers (terephthalic acid and ethylene glycol), allowing high recovery rates. However, the actual yield can be affected by factors such as the quality of the feedstock and the efficiency of the chemical reactions involved. Enzymatic recycling yields can vary based on several factors, including enzyme efficiency, substrate purity, and process conditions. In the aforementioned study, the base case scenario for enzymatic recycling reported an overall yield of ≈56%, taking into account various process efficiencies (e.g., calculated by multiplying 90% sorting yield, 93% flake, 95% pretreatment, 90% depolymerization, 79% total monomer, and 99% repolymerization). The authors consider that further research is needed to develop more environmentally friendly enzymatic PET hydrolysis processes, to improve enzyme efficiency, and also to improve feedstock preparation and product recovery.

This review focuses on recycling alternatives for two synthetic polymers widely used in the textile industry: PA and elastane. The recovery and recycling of these fibers is a major challenge. Different recycling approaches are described and a brief introduction of bio‐based PAs and elastane is given, as well as a description of recycling technologies that allow depolymerization by chemical routes and biochemical recycling. Thus, the aim of this review is to provide an overview of the existing recycling technologies and the application of bio‐recycling of these synthetic fibers.

## 2 Recycling and Degradation of Polyamides

PAs are semi‐crystalline polymers widely used in the textile industry, such as PA 6,6 (or Nylon 6,6) and PA 6 (or Nylon 6). PA 6,6 is produced by the condensation reaction of two monomers, adipic acid (AA) and hexamethylene diamine (HMDA).^[^ ^13^ ^]^

### 2.1 Thermo and Mechanical Recycling

**Figure 1:**
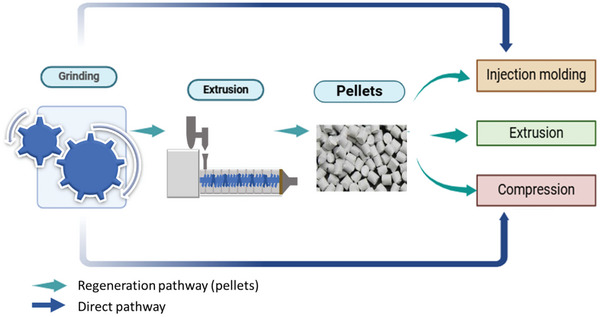
Mechanical recycling consists of shredding, melting, and molding. Shredding is referred to as powdering or pulverizing to reuse waste materials.

Mechanical recycling, also known as down‐cycling or open‐loop recycling, is a common technique in the textile industry.^[^ ^14^ ^]^ It involves processes such as grinding, melting, molding, granulating, and compounding to convert thermoplastic polymers into secondary raw materials for new applications^[^ ^15^ ^]^ (**Figure** **1**).

For mechanical recycling, the waste stream must be well cleaned and sorted to obtain high quality granules. The waste is not suitable for recycling and goes directly to incineration if it is contaminated by more than 15%.^[^ ^16^ ^]^ In general, mechanical recycling has some limitations. For example, the changes in the chemical structure and morphology of the material result in the modification of some physical and mechanical properties such as crystallinity index and tensile strength. Therefore, it is still limited by the degradation of material quality with each recycling cycle and the production of lower quality materials. However, for only cycle of recycling, mechanical properties similar to those of virgin materials can be maintained by thermo‐mechanical recycling.^[^ ^17^ ^]^

The nature of thermoplastic PA 6 and PA 6,6 allows them to melt (220 – 270 °C) and shaped into products using techniques like extrusion and injection molding, which can also be used for recycling.^[^ ^18^ ^]^ Thermo‐mechanical recycling uses a melt extrusion system which is the most used method for recycling PAs^[^ ^19^ ^]^ in textile industry.^[^ ^17^ ^]^ M.J. Lozano‐Gonzales and co‐workers^[^ ^20^ ^]^ investigated the injection molding recycling of PA 6 at 235 °C, they observed changes of 10–15% in the physical‐mechanical properties of PA 6 after 8 cycles. P. Xiu and co‐workers^[^ ^21^ ^]^ suggested that the incorporation of additives in the recycling process can avoid the loss of mechanical properties.

Thermo‐mechanical recycling can be applied to mixed wastes containing different thermoplastic polymers that are extruded into new hybrid fibers.^[^ ^22^ ^]^ The use of mechanical recycling can be economically viable in the context of a circular economy.^[^ ^23^ ^]^

### 2.2 Chemical Recycling

Chemical recycling is also known as tertiary or feedstock recycling. It involves the conversion of polymer chains into their molecular building blocks, such as oligomers and small molecules.^[^ ^24^ ^]^ A second generation polymer is produced from the recovered building blocks. Chemical recycling is associated with depolymerization reactions, which are typically endothermic processes that require energy input to break polymer chains into smaller molecular units^[^ ^25^ ^]^ and use elevated temperatures. For example, thermal cracking of PAs by pyrolysis varies depending on the specific process and desired results, but in general has been shown to degrade at 300 °C with significant degradation occurring at 400 °C.^[^ ^26^ ^]^ Therefore, the use of catalysts is often employed in depolymerization reactions to lower the activation energy and improve efficiency.^[^ ^27^ ^]^ Recently, R. Coeck and co‐workers^[^ ^28^ ^]^ have developed a novel depolymerization method of PA 6,6, a transamidation reaction that requires a catalyst by NH_3_ supported Nb_2_O_5_ and operates under conditions where the reaction temperature is 225 °C and 3 bar of NH_3_. PA 6,6 was completely depolymerized into monomers (94% of N,N’‐hexamethylene bis(acetamide)) and dimers (5% of N‐(6‐acetamidohexyl)adipamide).

An example of a chemical recycling process has been established for PA 6 to recover caprolactam, allowing the production of fibers with a quality comparable to virgin materials. The Resyntex project (2018) aims to use chemical recycling for various textile materials (cotton, polyester, Nylon, elastane, etc.) to recover the starting materials for synthetic fibers.^[^ ^29^, ^30^ ^]^

#### 2.2.1 Solvolysis

**Figure 2:**
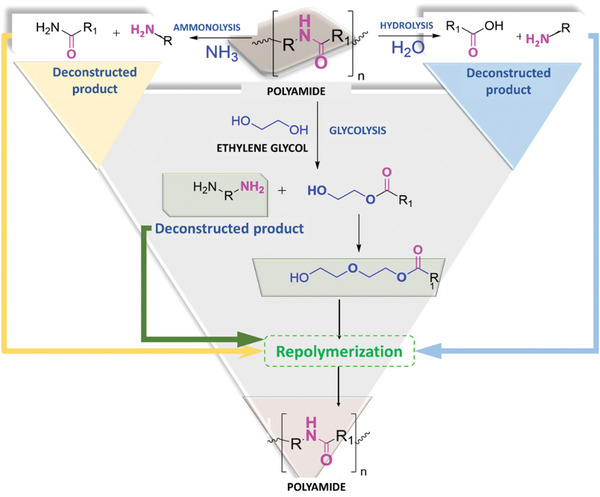
Solvolysis reactions for amide bonds polymers. The nucleophile reacts with the carbonyl group to give a cleaved product.

This method uses solvents such as water, methanol, ethylene glycol, and various amines and ammonium. Depolymerization reactions are performed using various molecules such as water or steam for hydrolysis,^[^ ^31^ ^]^ ammonium for ammonolysis,^[^ ^32^ ^]^ and alcohol/glycols for alcoholysis/glycolysis^[^ ^33^ ^]^ (**Figure** **2**).

PA recycling is primarily focused on hydrolysis. The chemical structure of PA 6,6 makes it more difficult to depolymerize than PA 6.^[^ ^34^ ^]^ It has also been reported that PA 6,6 can be completely hydrolyzed to adipic acid (AA) and hexamethylene diamine (HMDA) monomers using strong acids at high temperature and pressure.^[^ ^35^ ^]^ M. Polk and co‐workers^[^ ^36^ ^]^ demonstrated the depolymerization of PA 6,6, yielding 59.6% adipic acid and oligomeric mixtures with a molecular weight of 1434 g mol^−1^. Recently, R. Coeck and co‐workers^[^ ^28^ ^]^ demonstrated a transamination reaction that allows the depolymerization of PA 6,6 to easily processable monomers at relatively high concentrations. This method yielded ≈94% N,N'‐hexamethylene bis(acetamide) and 5% dimers. This approach offers a promising route to effective PA recycling. **Table** **1** summarizes solvolysis reactions catalyzed by acids, bases, or salts, primarily at high temperature and pressure.

**Table 1:** Summary of solvolysis reactions for polyamide.

Solvolysis method	Reagent/Substrate	Decomposition agent	Catalyst	Performance	References
Glycolysis	PA 6,6	Ethylene glycol	Diammonium hydrogen phosphate (2wt.%)	The molecular weight is ≈90 g mol^−1^	J. Datta et al., 2018^[^ ^31^ ^]^
Glycolysis	PA 6,6	Ethylene glycol	–	The molecular weight is ≈9000 g mol^−1^	K. J. Kim et al., 2006^[^ ^33^ ^]^
Amino‐glycolysis	PA 6,6	Ethylene glycol + Triethylene‐tetramine (TETA)	Diammonium hydrogen phosphate (2wt.%)	The molecular weight is ≈250 g mol^−1^	J. Datta et al., 2018^[^ ^31^ ^]^
Hydrolysis	PA 6,6	H_2_O	Acid hydrochloric (24.7%)	Yield recovery was 84%	S. Emik et al., 2018^[^ ^34^ ^]^
Acid hydrolysis	PA 6,6	HCl	–	Yield recovery was 72%	D. Patil et al., 2014^[^ ^35^ ^]^
Acid hydrolysis	PA 6	Formic acid, hydrochloric acid, and sulfuric acid	–	Yield recovery was 93%	S. Shukla et al., 2006^[^ ^37^ ^]^
Catalytic Hydrolysis	PA 6	Satured atmospheric steam	Benzyltrimethyl‐ammonium bromide	Isolation of adipic acid.	M B. Polk et al., 1998^[^ ^36^ ^]^
Acid hydrolysis	PA 6	Acetic anhydride (Ac_2_O)	DMAP (N,N‐dimethylamino‐pyridine)	78% of ε‐caprolactam	C. Alberti et al., 2019^[^ ^38^ ^]^

#### 2.2.2 Solvent‐Assisted Depolymerization

Solvent‐assisted depolymerization of PA 6,6 is critical to overcoming the challenges associated with its chemical recycling. **Table** **2** provides a summary of some examples of solvent assisted hydrolysis reactions. High temperature supercritical or subcritical solvents can promote intramolecular cyclization to form monomers.^[^ ^39^ ^]^ While supercritical liquid or superheated steam can depolymerize molten PA 6 at temperatures up to 420 °C, this method has not been successful for PA 6,6. During the hydrolysis of PA 6,6, the resulting reactive mixture of acids and amines tends to repolymerize to form a PA 6,6 resin.^[^ ^28^ ^]^

**Table 2:** Summary of solvent‐assisted depolymerization for PA.

Solvolysis method	Reagent/substrate	Decomposition agent	Catalyst	Performance	References
Ionic liquid assisted hydrolysis	PA 6	[Bmim]Cl		94% of Nylon degraded	J Chen et al., 2012^[^ ^39^ ^]^
Ionic liquid assisted hydrolysis	PA 6	[Emim][BF_4_]	DMAP (N,N‐dimethylamino‐pyridine)	62% of ε‐caprolactam	A. Kamimura et al., 2019^[^ ^25^ ^]^
Supercritical water assisted hydrolysis	PA6	Supercritical water	–	Yield recovery was 100%	M. Goto et al., 2006^[^ ^40^ ^]^
Supercritical water assisted hydrolysis	PA 6,6	Supercritical water	–	Nylon is completely degraded	L. Meng et al., 2004^[^ ^41^ ^]^
Microwave assisted hydrolysis	PA 6	37% HCl	–	90% of adipic acid and 86% of diamine	U. Cesarek et al., 2020^[^ ^42^ ^]^
Supercritical water assisted hydrolysis	PA 6	Subcritical water	Phosphotungstic heteropoly acid	78% of ε‐caprolactam	J. Chen et al., 2010^[^ ^43^ ^]^

#### 2.2.3 Hydrogenative Depolymerization

A. Kumar and co‐workers^[^ ^44^ ^]^ studied the hydrogenative depolymerization of PA 6. The study reports the first example of hydrogenative depolymerization of conventional PA, such as PA 6, using a ruthenium pincer catalyst. This process effectively breaks down tough polyamides into valuable monomers and oligomers. The process yielded significant amounts of 6‐amino‐1‐hexanol from PA 6, with conversion rates up to 99% under certain conditions. The authors achieved up to 99% conversion under optimized catalytic conditions. The yield of the product 6‐amino‐1‐hexanol from PA 6 was reported to be in the range of 32% to 55%, depending on the specific conditions and catalyst used. In their study, W. Zhou and co‐workers^[^ ^45^ ^]^ showed the hydrogenation yields for technical grade PA 6,6 under different conditions. The researchers evaluated various ruthenium (Ru) catalysts, focusing on Ru pincer complexes, which are known for their effectiveness in hydrogenation reactions. The hydrogenation reactions were carried out at elevated temperatures (up to 200 °C) and pressures (up to 100 bar of hydrogen). These conditions were crucial to increase the solubility of the polymers, which is a key factor influencing the reactivity of the substrate, the yield of diamine was 78% and the yield of diol was 62%.

The hydrogenation process is highlighted as a green sustainable and atom‐economical reaction that offers a promising direction for the chemical recycling of waste Nylon and potentially other PAs.^[^ ^44^, ^45^ ^]^

#### 2.2.4 Selective Dissolution and Precipitation

The dissolution and precipitation processes are also considered as part of the separation of polymer blends.^[^ ^46^ ^]^ This process can improve the recycling management of textile fibers. Recently, authors have demonstrated the selective dissolution of PA fibers based on the complexation and decomplexation of PA.^[^ ^47^ ^]^ The authors describe the use of a solvent by mixing calcium chloride water and ethanol (CEW).^[^ ^47^, ^48^ ^]^ With this solvent mixture, 80 to 90% of the PA precipitated with water can be obtained.

### 2.3 Biological Degradation

Biodegradation is the degradation of materials under biological conditions.^[^ ^49^ ^]^ It involves microorganisms and enzymes produced by bacteria, fungi, and yeasts that convert macromolecular chains into small organic molecules. These microorganisms then consume the small organic molecules as a carbon source under appropriate conditions.^[^ ^50^, ^51^ ^]^

To produce biomass and soil compost, biodegradation can occur either under aerobic conditions, producing carbon dioxide (CO₂) and water, or under anaerobic conditions, producing methane (CH₄) and water.^[^ ^52^ ^]^ However, not all synthetic polymers are directly consumed by microorganisms. Degradation depends on the chemical structure and physical properties of the polymers.^[^ ^53^ ^]^

**Figure 3:**
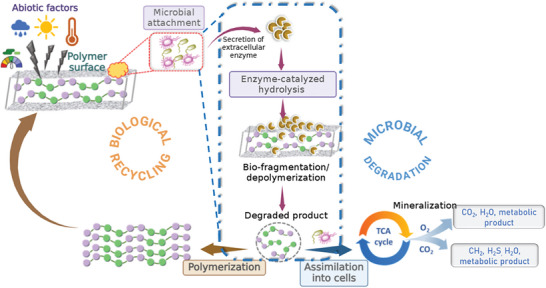
Mechanism of microbial degradation and biological recycling, microorganisms release extracellular secreted enzymes to degrade the polymer structure in the long term, then the degraded products or oligomers are assimilated into the cells being used as carbon source in the classical pathway to obtain energy. Biological recycling is a cycle in which the degraded products or oligomers are used to make second generation polymers (Design readapted from N. Mohanan et al., 2020^[^ ^87^ ^]^ and A. Magnin et al. 2020^[^ ^88^ ^]^).

The biodegradation process (**Figure** **3**) typically involves two main steps: a) cleavage of the polymer backbone and b) mineralization, i.e., the assimilation of small molecules (e.g., monomers, oligomers) by microbial cells.

In waste management, the concept of biodegradation can be divided into microbial degradation and enzymatic depolymerization. Microbial degradation depends mainly on the chemical structure. Microorganisms can attack the polymer backbone if it contains heteroatoms or C‐C double bonds. This process includes the mineralization step.^[^ ^87^ ^]^

In enzymatic depolymerization, the catalyzed reaction is influenced by physical properties such as hydrophobicity, degree of crystallinity, high molecular weight, functional groups, surface area, glass transition, and melting temperature, Young's modulus, presence of additives/plasticizers, and strength of C‐C bonds.^[^ ^54^, ^55^ ^]^ All of these properties can result in a very slow degradation process.

In the context of enzymatic recycling, degradation is the first step and is monitored by the mass loss of the synthesized polymer. The use of extracellular enzymes allows hydrolysis of the polymer backbone, releasing oligomers and monomers. In addition, isolated extracellular enzymes can potentially hydrolyze water‐insoluble polymers.^[^ ^56^ ^]^ The specificity of the enzymes makes them a useful tool for cleaving of low molecular weight degradation products.^[^ ^57^ ^]^

Thus, depolymerization can be used as a form of biological recycling, where monomers are reused for polymer synthesis.^[^ ^58^, ^59^ ^]^ A second generation of synthetic polymers can be assembled from the recovered monomers.

#### 2.3.1 Microbial Degradation

The chemical structure and physicochemical properties of PA make it difficult to degrade.^[^ ^60^ ^]^ The high intermolecular strength of hydrogen bonds in PA results in a slow degradation rate compared to polyesters.^[^ ^61^ ^]^ However, bacteria^[^ ^62^, ^63^ ^]^ and fungi^[^ ^64^, ^65^ ^]^ have been primarily used to study the microbial degradation of PA. However, no microorganism has been identified that can degrade the bulky industrial polymer PA 6,6 (Nylon 6,6).^[^ ^66^ ^]^

**Table** **3** shows a list of studies on the microbial degradation of PA 6,6 and PA 6. Most of the studies were conducted on linear or cyclic oligomers of PA (i.e., unwanted by‐products of PA production).

**Table 3:** Studies on PA 6,6 and PA 6 bacterial degradation.

Microbial communities	Substrates	Enzyme activity	Time of incubation	References
*Pseudomonas aeuruginosa NK87*	6‐Aminohexanoate	Hydrolase	–	K. Kanagawa et al., 1993^[^ ^67^ ^]^
*Pseudomonas aeuruginosa PAO1*	6‐Aminohexanoate	Cytochrome c oxidase	20h	I. D. Prijambada et al., 1995, B. W. Holloway et al., 1986^[^ ^68^ ^]^
*Flavobacterium sp K175*	6‐Aminohexanoate	Hydrolase	16 h	S. Kakudo et al., 1993^[^ ^69^ ^]^
*Agromyces sp*. *(Strain KY5R)*	6‐Aminohexanoate	Hydrolase	7 days	K. Yasuhira et al., 2007^[^ ^70^ ^]^
*Geobacillus thermocatenulatus*	PA 6,6	unknown	20 days	K. Tomita et al., 2003^[^ ^61^, ^71^ ^]^
Marine bacterias (*Bacillus cereus, Bacillus sphericus, Vibrio furnisii et Brevundimonas vesicularis*)	PA 6, PA 6,6	unknown	3 months	M. Sudhakar et al., 2007^[^ ^72^ ^]^
*Corynebacterium aurantiacum*	PA 6	unknown	48 h	T. Fukumura, 1966^[^ ^73^ ^]^
*Arthrobacter citreus*	ɛ‐caprolactam	unknown	7 days	N. N. Baxi et al., 2019^[^ ^74^ ^]^
*Anoxybacillus rupiensis Ir3*	PA 6	unknown	7 days	M. Mahdi et al., 2016^[^ ^75^ ^]^
*Achromobacter guttatus KI72*	PA 6	unknown	48 h	S. Kinoshita et al., 1975^[^ ^76^, ^77^ ^]^
*Pseudomonas jessenii f*	Caprolactam	unknown	7 days	P. Marleen Otzen et al., 2018^[^ ^78^ ^]^
*Acinetobacter sp strain 6*	6‐Aminohexanoate cyclic dimer	Amidase/esterase	–	Y. L. Wei et al., 2003^[^ ^79^ ^]^

The white rot fungus IZU‐154 was shown to degrade PA by oxidative metabolism using a peroxidase, reducing the molecular weight from 84000 g mol^−1^ to 5500 g mol^−1^ in 20 days.^[^ ^62^ ^]^ M. Fujisawa and co‐workers^[^ ^80^ ^]^ showed the degradation of PA 6,6 using a laccase‐mediated system (LMS) with the mediator 1‐hydroxybenzotriazole (HBT), reducing the molecular weight from 79 300 g mol^−1^ to 14 700 g mol^−1^. **Table** **4** lists the major fungal species involved in PA degradation.

**Table 4:** Studies reporting PA 6,6 and PAs degradation by fungi.

Species/strains	Substrates	Enzyme activity	Time of incubation	References
*Trametes versicolor NCIM 1086*	PA 6	Manganese peroxidase	90 days	S. Chonde et al., 2012^[^ ^81^ ^]^
*Trametes versicolor*	PA 6,6	Peroxidase	3 days	M. Fujisawa et al., 2001^[^ ^80^ ^]^
*Bjerkandera adusta*	PA 6	Manganese peroxidase and lignin peroxidase activities	60 days	J. Friedrich et al., 2005^[^ ^65^ ^]^
*Phanerochaete chrysosporium*	PA 6	Unknown	5 months	U. Klun et al., 2003^[^ ^64^ ^]^
*Aspergillus niger*	PA	Unknown	2 months	M. S. Marques et al., 2000^[^ ^82^ ^]^
*Aspergillus niger AF3*	PA 6	Unknown	3 weeks	H. A. Sanuth et al., 2013^[^ ^83^ ^]^
*White rot fungi*	PA 6,6	Manganese peroxidase (MnP)		S. Negoro et al., 1983^[^ ^63^ ^]^
*White rot fungi IZU‐154, Phanerochaete chrysosporium (ATCC 34 541) and Trametes versicolor (IFO 7043)*	PA 6,6	Manganese peroxidase (MnP)	20 days	T. Deguchi et al., 1993^[^ ^62^ ^]^
*Fusarium solani, F. oxysporum*	4		28 days	K. Tanibacha et al., 2010^[^ ^84^ ^]^

a: PA derived from tartaric acid and hexamethylenediamine.

#### 2.3.2 Enzymatic Degradation

The process of hydrolytic degradation is typically mediated by extracellular enzymes secreted by microorganisms. Once microorganisms attach to the polymer surface, a heterogeneous enzymatic attack occurs. This attack can be initiated at the chain ends (exo attack) or in the middle of the chain (endo attack).^[^ ^85^ ^]^

However, enzymatic degradation basically occurs in two steps^[^ ^57^, ^86^ ^]^: i) the enzyme binds to the polymer substrate,^[^ ^57^ ^]^ ii) followed by bond fragmentation, where a reverse process of polycondensation occurs and polymer bonds of the hetero chain are fragmented.^[^ ^87^ ^]^

When enzymes adhere to the polymer surface, hydrolytic attack occurs primarily in the amorphous regions of the polymer surface layer,^[^ ^63^ ^]^ which are more susceptible to enzymatic attack than the crystalline regions.^[^ ^49^, ^88^ ^]^ Upon enzymatic attack, some physicochemical properties of the polymer change slightly, such as the degree of crystallinity, thermal stability, and polydispersity index.^[^ ^89^ ^]^ This is related to water diffusion into the polymer, which is influenced by parameters such as porosity, crystallinity, surface roughness, hydrophobicity, and sample size.^[^ ^90^ ^]^ Hydrolytic attack on the polymer surface allows the release of oligomers or low molecular weight molecules.^[^ ^91^ ^]^ The cleavage of the polymer backbone involves the breakdown of the polymer into small fragments such as oligomers and monomers, making enzymatic hydrolysis a heterogeneous process.^[^ ^91^ ^]^ Therefore, the polymer surface morphology can follow two types of degradation phenomena: surface erosion and bulk degradation.

The surface erosion phenomenon can be monitored by mass loss, polymer swelling, and changes in morphology and molecular weight.^[^ ^92^ ^]^ The bulk erosion phenomenon usually occurs when water molecules penetrate into the polymer network. Thus, enzymatic hydrolysis can occur in the internal chains.^[^ ^93^ ^]^ These two phenomena differ in terms of the constant rate of erosion. Bulk erosion will not have a constant erosion rate. Thus, the mass lost will spontaneously be more than half of its mass.^[^ ^94^ ^]^ The potential of enzymes for modification and surface functionalization of polymers has been well studied.^[^ ^56^, ^95^, ^96^, ^97^, ^98^, ^99^ ^]^ The functional groups of PAs can be modified or hydrolyzed by enzymes.^[^ ^100^ ^]^ In **Table** **5**, different studies have investigated the surface hydrolysis of PA fibers to increase hydrophilicity and improve the dyeing process in the textile industry). The surface modification is monitored by the degree of water absorption (wettability), the K/S value, and the ionic groups on the surface.^[^ ^95^, ^101^, ^102^ ^]^ This modification does not directly affect the mechanical properties of the bulk polymer.^[^ ^99^ ^]^ The backbone of the PA chain contains amide linkages that are hydrolyzed by proteases such as subtilisin^[^ ^103^ ^]^ or cutinases,^[^ ^104^ ^]^ and amidases (acylamidases).^[^ ^56^ ^]^

**Table 5:** Summary of Nylon degrading enzymes for surface modification.

Enzyme	Species/strains	Substrate	Performance	Soluble degraded product	References
Protease	*Beauveria sp*	PA adipic acid bishexyl‐amide	Surface modification	AA	S. Heumman et al., 2006^[^ ^105^ ^]^
Polyamidase	*Beauveria brongniartii*	PA 6 and 6,6	Surface modification	AA	E. Almansa et al., 2008^[^ ^97^ ^]^
Protease	*Bacillus subtilis*	PA 6,6	Surface modification	Amines	M. Kanelli et al., 2017,^[^ ^99^ ^]^ C. Silva et al., 2007^[^ ^96^ ^]^
Aryl acylamidase	*Nocardia farcinica*	Adipic acid bishexylamide	Surface modification	Hexanoamide	S. Heumman et al., 2009^[^ ^56^ ^]^
Cutinase	*Fusarium solani*	PA 6	Structure modification	Amines	Silva et al., 2005^[^ ^104^ ^]^

S. Negoro and co‐workers^[^ ^106^ ^]^ identified hydrolytic enzymes within the amidase signature family, such as 6‐aminohexanoate cyclic dimer hydrolase (NylA, EC 3.5.2.12), 6‐aminohexanoate dimer hydrolase (NylB, EC 3.5.1.46), and 6‐aminohexanoate oligomer endo‐hydrolase (Nylon hydrolase) (NylC, EC 3.5.1.117). These enzymes were mainly studied in the hydrolysis of linear and cyclic Nylon oligomers, which are by‐products of the polymerization of PA 6. A few years later, S. Negoro and co‐workers^[^ ^63^ ^]^ developed a thermostable mutant enzyme (NylCp2) with four mutations introduced by site‐directed mutagenesis (D122G, H130Y, D36A, E263Q). This enzyme showed good degradability at 60 °C, but was not suitable for depolymerization due to low activity. H. Puetz and co‐workers^[^ ^107^ ^]^ improved a nylonase (NylCTS) by a single round of random mutagenesis to develop a high‐throughput screening (HTS) system for direct evolution. **Table** **6** summarizes recent studies on nylonases.

**Table 6:** Summary of Nylon degrading enzymes.

Enzyme	Species/strains	Substrate	Performance	Soluble degraded product	References
Manganese peroxidase	*Fungus strain IZU‐154*	PPA 6,6	Hydrolytic partial degradation	Amines	T. Deguchi et al., 1998^[^ ^108^ ^]^
Laccase	*Trametes versicolor*	PA 6,6	Oxidative degradation	Oligomers	M. Fujisawa et al., 2001^[^ ^109^ ^]^
6‐Aminohexanoate oligomer endo‐hydrolase	*Flavobacterium sp. KI72*	PA 6	Hydrolytic degradation	Polyamide 6 linear oligomer	S. Kakudo et al 1993^[^ ^110^ ^]^
6‐Aminohexanoate oligomer exo‐hydrolase	*Flavobacterium sp. KI72*	PA 6 linear oligomer	Hydrolytic degradation	6‐Aminohexanoate	S. Kinoshita et al., 1981^[^ ^111^ ^]^
6‐Aminohexanoate‐cyclyc dimer hydrolase	*Acromobacter guttatus KI72*	6‐aminohexanoate cyclic dimer	Hydrolytic degradation	6‐Aminohexanoate‐linear dimer	S. Kinoshita et al., 1977^[^ ^77^, ^111^, ^112^ ^]^
6‐Aminohexanoate‐linear dimer hydrolase	*Flavobacterium K172*	6‐aminohexanoate linear dimer	Hydrolytic degradation	6‐Aminohexanoate	S. Negoro et al., 2007^[^ ^113^ ^]^
Endo‐type 6‐aminohexanoate‐ oligomer hydrolase NylCp2 (D122G/H130Y/D36A/E263Q)	*Arthrobacter sp. (plasmid pOAD2‐encoding enzyme)*	PA 6 linear oligomer	Hydrolytic degradation	6‐Aminohexanoate	S. Negoro et al., 2012^[^ ^63^ ^]^
Endo‐type 6‐aminohexanoate‐ oligomer hydrolase NylC_TS_ (P27Q/F301L)	*Arthrobacter sp. (plasmid pOAD2‐encoding enzyme)*	PA 6	Depolyme‐rization	6‐Aminohexanoate	H. Puetz et al., 2023^[^ ^107^ ^]^
Endo‐type 6‐aminohexanoate‐ oligomer hydrolase NylC_K_‐TS (NylC_K_‐S111G/A137L) and NylC_A_‐S111G/A137L/E263Q)	*Kokuria sp*.	PA 6	Depolyme‐rization	6‐Aminohexanoate	E. Bell et al., 2024^[^ ^114^ ^]^
NylB‐SCY (R187S/F264C/D370Y)	*Arthrobacter sp.K172*	PA 6	Depolyme‐rization	6‐Aminohexanoate	E. Bell et al., 2024^[^ ^114^ ^]^

Recently, E. Bell and co‐workers^[^ ^114^ ^]^ studied the temperature‐dependent activity of a nylonase C. Comparative time‐course reactions incubated at 40–70 °C showed enzyme‐dependent variations in product distributions and the extent of PA 6 film depolymerization. Significant Nylon degradation activity was rare within this temperature range. The researchers identified a thermostabilized variant of NylCK, an N‐terminal nucleophile (Ntn) hydrolase, named NylCK‐TS. This variant has a melting temperature (Tm) of 87.4 °C, which is 16.4 °C higher than the wild‐type enzyme. Using NylCK‐TS, the authors demonstrated a hydrolysis yield of 0.67 wt.% for a PA 6 film.

J. de Witt and co‐workers^[^ ^115^ ^]^ successfully identified three novel nylonases (NylC1, NylC2, and NylC3) through library screening and in silico analysis. These novel enzymes show different sequence identities (ranging from 84% to 32%) compared to the previously characterized NylCp2 from *Paenarthrobacter ureafaciens*. However, the authors confirmed the activity of the novel nylonase candidates toward cyclic PA oligomers by the detection of soluble degradation products. These nylonases were also active on synthesized poly (ester amides) (PEA) when combined with cutinase (LCC), resulting in the hydrolysis of ≈1% of the total polymer. These results increase the diversity of nylonases for PA and poly(esteramide) (PEA) recycling processes and the potential for future enzyme engineering.

The current challenges in enzymatic depolymerization cannot effectively solve the recycling problem. Various studies on nylonases have shown very low yield conversions, indicating that further research is needed to achieve complete hydrolysis. Most studies have focused on PA 6 depolymerization because it is less crystalline than PA 6,6. Recent research has emphasized enzyme engineering to develop more efficient and thermostable enzymes for PA depolymerization. Thus, recent findings demonstrate that enzyme engineering is a tool to improve biocatalysts and the ability to recycle materials such as PA. However, the design of efficient enzymes still needs to be developed so that enzymatic depolymerization can be considered as a competitive method to mechanical and chemical ones.

## 3 Recycling Alternatives for Elastane

Elastane is a polyurethane (PU) elastomer that is widely used in the textile industry. The best known is Lycra (Invista), also known as elastane or Spandex, which is a technical term used to describe this PU as a polyether‐polyurea copolymer.^[^ ^116^ ^]^ Other brand names include Elaspan (Invista), Acepora (Taekwang Industrial), Creora, Linel (Fillattice), Dorlastan and Roica (Asahi kasei), and ESPA (Toyobo Co).^[^ ^22^, ^117^ ^]^

Unlike other thermoplastic polymers, PU is a multiblock copolymer with a complex structure consisting of: soft segments composed primarily of polyols (e.g., polyether polyol, polyester polyol, or polycarbonate polyol); hard segments composed of diisocyanates; and finally a chain extender, typically a short‐chain glycol.^[^ ^118^ ^]^ Because of their multiblock copolymer structure, PUs can be depolymerized and recycled. However, the complex chemical structure of PU elastomers makes them difficult to degrade. Recycling can be associated with degradation, which occurs with all types of thermoplastic polymers.

The choice of recycling process depends on the desired product. Physical and mechanical properties can be affected during recycling, especially in injection molding where different cooling conditions affect mechanical properties.^[^ ^119^ ^]^

### 3.1 Mechanical Recycling

Mechanical recycling is the simplest and most basic way to recycle PU. However, thermo‐mechanical recycling is a promising method for recycling PU using twin‐screw extrusion. This method can be used in a continuous recycling process.^[^ ^119^, ^120^ ^]^

N. Vidakis and co‐workers.^[^ ^121^ ^]^ demonstrated a thermo‐mechanical process for recycling thermoplastic polyurethane (TPU), where six repetitions of thermal cycles were feasible. Further increases in thermal cycles caused processability problems. A study on the recycling of used boots was carried out by A. Nanni and co‐workers,^[^ ^122^ ^]^ who showed that it is possible to recycle TPU recovered from new boots up to three cycles. They observed a loss of tensile strength (from 44 to 26 MPa) and elongation at break (from 1046% to 840%).

B. Wölfel and co‐workers^[^ ^119^ ^]^ emphasize that while the molar mass decreases significantly with each recycling cycle, it stabilizes at an asymptote, indicating that there is a limit to how much the material can be degraded by mechanical recycling. Mechanical properties such as tensile strength and elongation at break also change, reflecting the effect of recycling on the performance of the material. **Table** **7** provides an overview of the literature on thermal and mechanical recycling, including polymer types, reinforcing materials, and processing methods.

**Table 7:** Review of related thermal and mechanical recycling.

TPU grade	Blended materials	Processing method	Recyclability	Reference
Ravathane	No materials	Extrusion single screw	Viable after 6 cycles	N. Vidakis et al., 2023^[^ ^121^ ^]^
Polyether‐based TPU resin of 80A Shore hardness	Polyether‐based thermoplastic PU	Twin screw extruder	8 recycling steps	B. Wölfel et al., 2020^[^ ^119^ ^]^
Boots	TPU and Polypropylene (PP)	Grinded	3 cycles	A. Nanni et al., 2023^[^ ^122^ ^]^

Today, mechanical recycling is the most common alternative for reusing. The materials produced from mechanically recycled PU waste are low‐value products with limited applications.^[^ ^123^, ^124^ ^]^

### 3.2 Chemical Recycling

#### 3.2.1 Solvolysis

**Figure 4:**
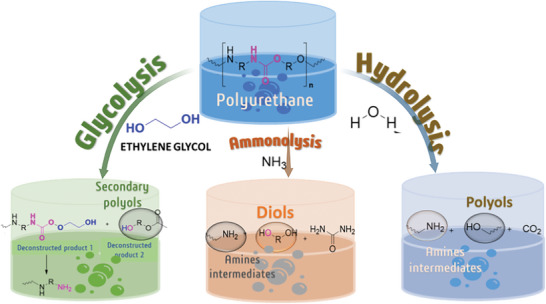
Major solvolysis process for PU depolymerization.

The solvolysis process for PU is primarily focused on the recovery of polyols for reuse in the production of a second generation of PU. Polyols are a key component in the formulation of PU.^[^ ^125^ ^]^ **Table** **8** summarizes the most commonly used solvolysis processes for PU recycling including: hydrolysis,^[^ ^126^ ^]^ glycolysis,^[^ ^127^ ^]^ ammonolysis,^[^ ^128^ ^]^ and alcoholysis.^[^ ^129^ ^]^ These processes have been reported for the recovery of polyols and amines^[^ ^130^ ^]^ (**Figure** **4**). Among these, glycolysis has received significant attention due to its industrial applications.^[^ ^125^, ^131^, ^132^ ^]^ The glycolysis process involves a transesterification reaction in which the ester group attaches to the carbonyl carbon of the urethane and is then exchanged with the hydroxyl group of the glycol.^[^ ^133^ ^]^

**Table 8:** Summary of some PU solvolysis reactions.

Solvolysis method	Substrate	Decomposition agent	Catalyst	Performance	Reference
Glycolysis	PU elastomer	Ethylene glycol (EA)	Diethanolamine (DEA)		J. Borda et al., 2000^[^ ^127^ ^]^
	PU elastomer	Diethylene glycol (DEG)	AcK (Potassium acetate)	The remaining products yield was 87–93%	C.‐H. Wua et al., 2003^[^ ^136^ ^]^
	PU elastomer based on polyester polyol	Crude glycerine of 60% purity	–	The product yield obtained was 700–1020 g mol^−1^	P. Kopczynska et al., 2016^[^ ^137^ ^]^
Glycolysis‐hydrolysis	PU and dicarbamate	Ethylene glycol	Sodium Acetate	The yield of amine recovered after glycolysis‐hydrolysis is 30%	P. Zahedifar et al., 2021^[^ ^81^ ^]^
Methanolysis	TPU	Subcritical methanol		Recovery of 4,4′‐methylene diphenyl carbamate (MDC), 1,4‐butanediol (BDO) and dimethyl adipate (DMA)	Liu.L et al., 2017^[^ ^118^ ^]^
Base‐catalyzed transcarbamoylation	TPU	MeOH (methanol)/THF	Tert‐butoxide (t‐BuOK)	86% of diisocyanates recovered	L. Zhao et al., 2021^[^ ^139^ ^]^
Hydrolysis	Polycarbonate‐polyurethane	Deep eutectic solvent (Choline chloride:urea)	–	Recovery of 100% (PCDL) and 57% of (o‐toluidine)	H. Zhang et al., 2020^[^ ^140^ ^]^
	Poly(ether‐urethane)	Perchloric acid (HClO_4_) (60%)	–	Hard segmented recovery of 99 wt.%	H. Suzuki et al., 1970^[^ ^141^ ^]^
	PU foams	Succinic, phthalic, or adipic dicarboxylic acids		Recovery of polyols	N. Gama et al., 2021^[^ ^126^ ^]^

However, there are few examples of depolymerization of PU elastomers by solvolysis. For PU foam, glycolysis has been reported as the most common method in the industry^[^ ^131^, ^132^, ^134^ ^]^ including the use of catalysts such as organic bases (e.g., ethanolamine and diethanolamine),^[^ ^135^ ^]^ octanoates, organic acids and acetates.^[^ ^126^ ^]^ For example, L. Ramirez and co‐workers^[^ ^24^ ^]^ demonstrated the recycling of elastomeric PU waste by the glycolysis process, using ethylene glycol (EG) (POCH) as the glycolysis agent and potassium acetate (KAc) (POCH) as the catalyst.

In comparison, other processes such as acidolysis, hydrolysis, and aminolysis have a carbon efficiency of ≈85% because they produce some by‐products such as CO_2_ amines and amides in addition to the repolyol. The atom economy for these processes is also relatively high, ranging from 92% to 94%. Overall, glycolysis stands out as the most efficient method in terms of yield and product quality, making it a promising candidate for chemical recycling of PU waste.^[^ ^131^ ^]^

#### 3.2.2 Solvent‐Assisted Depolymerization

The application of intensification processes such as supercritical and subcritical fluids have also been studied as a method of recycling polymers in order to obtain monomers. PU elastomer can be treated in subcritical methanol as a result, three monomers are obtained 4,4′‐methylene diphenyl carbamate (MDC), 1,4‐butanediol (BDO) and dimethyl adipate (DMA), but in supercritical methanol, some undesirable by‐products, including amines, tetrahydrofuran (THF) and macrolides, are obtained.^[^ ^118^ ^]^

#### 3.2.3 Hydrogenative Depolymerization

The depolymerization of TPU foams has also been studied by catalytic hydrogenation in the presence of ruthenium [Ru] complex, which leads to the cleavage of the C‐N bond of amide and carbamate groups. In this process, 76% of the diamines and 85% of the diols were recovered.^[^ ^45^ ^]^

#### 3.2.4 Selective Dissolution

Elastane is a fiber that is found in many different apparel fabrics today, making it difficult to recover from other blended fibers. Therefore, different organic solvents have been evaluated in order to apply a selective solvent for elastane to separate it from another fiber. Selective separation of elastane by dissolution/precipitation method has been studied by L. Vonbrül and co‐workers, the mixing of N,N‐dimethylformamide (DMF) and tetrahydrofuran (THF) in a specific ratio can be carried out at room temperature.^[^ ^142^ ^]^ As a part of the recycling process, elastane can be removed from blended fabrics by dissolving it in solvents such as N,N‐dimethylformamide (DMF).^[^ ^143^ ^]^ The dissolution of elastane by the use of DMF has been reported at the optimum operating conditions of 70 °C for 4 h.^[^ ^144^ ^]^ The non‐hazardous solvent dimethyl sulfoxide (DMSO) is also selected as a suitable selective solvent for elastane at conditions of 120 °C for 5 to 10 min.^[^ ^145^ ^]^

Recently, green solvents have been investigated for the selective dissolution of elastane. They are being used as a recycling method for mixed textile fibers.^[^ ^146^ ^]^ The authors describe bio‐based solvents such as δ‐valerolactone and tetrahydrofurfuryl alcohol as potential solvents for dissolving elastane from textile blends such as elastane/polyester, elastane/P, and elastane/cotton.

### 3.3 Biological Degradation

#### 3.3.1 Microbial Degradation

PU has a complex chemical structure that is difficult to degrade in compost and soil conditions.^[^ ^147^ ^]^ It is also designed to withstand environmental factors such as climatic conditions (e.g., low and high temperatures, humidity), abrasion, and microbial attack (biotic and abiotic degradation).^[^ ^58^ ^]^ However, microbial degradation of PUs has mostly been carried out on a polyester urethane using fungi,^[^ ^147^, ^148^, ^149^ ^]^ bacterial,^[^ ^88^, ^150^ ^]^ and enzymatic hydrolysis (e.g., polyurethanases).^[^ ^151^ ^]^ Degradation of polyether urethane is less common and is usually performed by fungal species.^[^ ^152^ ^]^

Degradable PUs are obtained by incorporating labile and hydrolyzable moieties into the polymer chain.^[^ ^153^ ^]^ Unlike most synthetic polymer materials, PU can be susceptible to microbial attack.^[^ ^150^ ^]^ For example, polyester polyurethanes are more susceptible to microbial degradation than the polyether urethanes.^[^ ^150^ ^]^ It has also been reported that the resistance of polyether polyurethanes to biodegradation mechanisms is due to difficulties in exo‐depolymerization, whereas the resistance of polyester polyurethanes is due to endo‐depolymerization.^[^ ^154^ ^]^ Studies on polyester hydrolysis have been reported, showing two types of lipases that exhibit a difference in the mechanism of enzymatic degradation, such as endo‐ and exo‐cleavage routes.^[^ ^155^ ^]^

In addition, in recent years, the susceptibility of poly(ester urethanes) and poly(ether urethane ureas) to biodegradation has been a subject of great interest in biomedical applications.^[^ ^156^, ^157^ ^]^ In the textile industry, elastane is one of the most widely used synthetic polymers in blended textile fibers, but there is limited information on the microbial degradation of poly(ester‐ether) urethane copolymers.

#### 3.3.2 Enzymatic Depolymerization

In recent years, only a few studies have reported the enzymatic depolymerization of TPU.^[^ ^58^, ^59^, ^158^, ^159^ ^]^ Recently, A. Magnin and co‐workers^[^ ^58^ ^]^ reported the enzymatic depolymerization of polycaprolactone polyol polyurethane hydrolyzed with an esterase and amidases. The degraded products after hydrolysis were 6‐hydroxycaproic acid and 4,40‐methylenedianiline. The chemical structure of PU elastomer is based on poly(ester‐ether) urethane or poly(ester‐ether) urea, which makes it a very complex structure for enzymatic degradation.^[^ ^160^ ^]^ In general, hydrolysis of TPU allows the release of polyols and diisocyanates. To our knowledge, no studies have been reported on the enzymatic depolymerization of elastomeric PAs.

However, many studies related to the enzymatic degradation of PA elastomers are mostly applied to tissue regeneration in the field of biomedicine. In general, cell‐derived enzymes such as CE (cholesterol esterase), elastase and carboxylesterase have been shown to hydrolyze soft segments of polyether urethane and polyester urethane.^[^ ^161^, ^162^, ^163^, ^164^ ^]^ Therefore, several active enzymes have been reported to degrade PU substrates by cleavage of functional groups such as ester bonds by esterase^[^ ^161^, ^165^ ^]^ and lipases,^[^ ^155^ ^]^ amide bonds by proteases from fungi and bacteria microorganisms^[^ ^154^, ^161^, ^166^, ^167^ ^]^ and urethane bonds by ureases.^[^ ^58^ ^]^ **Table** **9** provides a brief summary of enzymatic degradation. For example, commercially available *Candida rugosa* lipase has been successfully used to degrade synthetic poly(ester‐urethane) particles in an aqueous medium.^[^ ^168^ ^]^ It has also been reported that polyamidases (from *Nocardia farcinica*) and other amidases can cleave the urethane bond in PU polyesters with different degrees of crystallinity.^[^ ^169^ ^]^ Other enzymes, such as proteinase K, showed degradative capacity when treated with a TPU/PLA polymer blend.^[^ ^170^ ^]^ However, enzymatic hydrolysis of poly(ether urethane) elastomers can be difficult. S. Hsu and co‐worker^[^ ^171^ ^]^ reported an oxidative pretreatment of polyether urethane with H_2_O_2_ and CoCl_2_ prior to enzymatic attack. The authors used plant derived enzymes such as papain for hydrolysis.

**Table 9:** Summary of the enzymatic degradation of a TPU.

Enzyme	Microorganism strain/origin	Substrate	Incubation time	Performances	References
Cutinase	*Humicola insolens*	Poly(ester‐urethane)	7 days	Decrease of the average molecular weight by 84% and 42%	F. Di Bisceglie et al., 2022^[^ ^172^ ^]^
Lacasse (mediator system)	*Trametes versicolor*	PCL and PTHF‐based PU	18 days	Decrease in molar mass	Magnin et al., 2021^[^ ^173^ ^]^
Cholesterol esterase (CE)	Bovine pancreas	Poly (carbonate urethane) (PCU) and poly(ether urethane) (PEU)	36 days	Change of molecular weight	Christenson et al., 2006^[^ ^158^ ^]^
Urease	*Canavalia ensiformis*	Poly (ester‐urethane‐urea)	34 days	No significant impact	C. Borrowman et al., 2020^[^ ^174^ ^]^
Esterase	*Bacillus subtilis*	Poly (ester‐urethane‐urea)	34 days	Reduction of molecular weight	C. Borrowman et al., 2020^[^ ^174^ ^]^
Esterase/ Polyurethanase	*Comamonas acidovorans*	poly(ester) urethane (Impranil)	3 days	Change of molecular weight	A. B. Allen et al., 1999^[^ ^175^ ^]^
Esterase	*Alicycliphilus sp. BQ8*	Poly (ester urethane)	15 days	Degradation of ester bonds	Perez‐Lara et al., 2016^[^ ^176^ ^]^
Lipase	*Candida antarctica*	Poly (ester‐urethane)	72h	Cyclic oligomers	H. Hayashi H. et al., 2011^[^ ^130^ ^]^
Lipase	*Candida rugosa*	Poly (ester)urethane (Impranil)	NM	Diethylene glycol	R. Gautam et al., 2007^[^ ^168^ ^]^
Lipase	*Rhizopus delemar*	Poly(ester‐urethane)	70 h	53% degradation of the original polyurethane film	Tokiwa et al., 1988^[^ ^155^ ^]^
Lipase	*Candida antarctica, Mucor miehei, Pseudomonas cepacia, Pseudomonas fluorescens*	poly(ester‐urethane)	24h	Molecular weight less than 500 g/mol	T. Takamoto et al., 2001^[^ ^177^ ^]^
Alpha‐chymotrypsin	Bovine type II	Poly (ester urea)	28 days	Decrease of molecular weight	G. A. Skarja et al., 2001^[^ ^178^ ^]^
Alpha‐chymotrypsin	Bovine pancreas	Poly (ether urethane)	15 days	Decrease of molecular weight	R. Smith et al., 1987^[^ ^179^ ^]^

NM: Not mentioned in the study

Enzymatic recycling of PU elastomers remains a challenge for further enzyme research. Currently, enzymes such as cutinase (or polyester hydrolases) from *Humicola insolens* have shown promising results for hydrolysis on the polyester fragment of PU.^[^ ^172^ ^]^ In addition to hydrolytic enzymes, oxidases such as laccase in a mediator system may be an alternative for TPU degradation.^[^ ^173^ ^]^ A chemoenzymatic approach was used to convert poly(ether)polyurethane in two steps, first conversion of dicarbamates by glycolysis and then hydrolysis of dicarbamates to small molecules using an enzyme identified from a metagenome library, the urethanase UMG‐SP‐2. The yield of enzymatic hydrolysis for PU monomers, specifically using urethanase UMG‐SP‐2, achieved ≈65% conversion to toluene diamine (TDA) within 24 h. Further addition of enzyme resulted in full conversion after 48 h. This indicates that the enzymatic process can effectively convert low molecular weight dicarbamates to aromatic diamines, which are valuable for the synthesis of new PUs.^[^ ^180^ ^]^

Recently, K. Xin and co‐workers^[^ ^181^ ^]^ identified an amidase GatA250 enzyme that cleaves only urethane bonds in polyester‐polyurethane, but the degradation efficiency was very low. However, when the amidase GatA250 was introduced with Cutinase LCC, which has high hydrolytic activity on the ester bonds, the degradation efficiency was higher for PU film (42.2%) and foam (13.94%).

## 4 How Sustainable is Recycling Polyamide and Elastane?

PA‐based waste poses some environmental problems, such as microplastic pollution in aquatic environments (from fishing nets and synthetic textile fibers from laundering). L. Zheng and co‐workers^[^ ^182^ ^]^ emphasize the need for improved recycling technologies and methods to effectively address the environmental impact of PA waste. The authors also point to the use of bio‐based PAs as a viable method to reduce dependence on limited petrochemical resources and promote sustainability in polymer production. However, it is important to note that not all bio‐based PAs are biodegradable. Currently, only PAs derived from PA‐4 and itaconic acid are reported to be biodegradable.^[^ ^183^, ^184^ ^]^

In general, the most common method of recycling PA waste is mechanical recycling. This involves grinding the waste materials and then reprocessing them by methods such as extrusion and injection molding.^[^ ^20^ ^]^

However, mechanical recycling often results in to performance degradation of the recycled material, especially when the recycled content is high.

N. Van der Velden and co‐workers^[^ ^185^ ^]^ emphasize the need for further studies on life cycle inventory data of textile products in order to reduce the variability of LCA results and improve the accuracy of the environmental assessment. As PA is not the most environmentally friendly material, the manufacturing process can reduce its impact. The study suggests that improvements in this area could improve the sustainability of PA textiles. **Table** **10** compares mechanical and chemical recycling of PA from a sustainability perspective.

**Table 10:** Comparison of mechanical and chemical recycling of PA fibers.

Sustainability aspect	Mechanical recycling	Chemical recycling
Environmental Impact	‐ Lower energy consumption ‐ Contamination issues ‐ Degradation of properties ‐ Lower waste generation	‐ Varies by process (solvolysis, hydrogenation, dissolution) ‐ Can be lower energy ‐ Handles wider range of feedstock ‐ Potential for higher‐quality monomers ‐ Hazardous byproducts possible
Efficiency and yield	‐ Efficiency decreases with contamination ‐ Yield can be affected by contamination	‐ Efficiency varies by process ‐ Can achieve higher yields ‐ Requires more complex processing and energy inputs
Scalability	‐ Well‐established ‐ Easier to scale and implement ‐ Existing infrastructure can be adapted	‐ Still in early development phase ‐ Challenges in scaling up
Commercial viability	‐ High market demand ‐ Economically viable	‐ Growing market demand for high‐value applications ‐ Economically viable for high‐value applications

Elastane is a synthetic fiber made of PU, which makes it difficult to recycle. PU differ in structure and depend on the combination of hard and soft segments or different stoichiometric ratios (NCO: OH). PU is a polymer formed by the reaction of OH (hydroxyl) groups of polyols with NCO (functional isocyanate) groups of isocyanates.^[^ ^93^, ^186^ ^]^ Elastane is often blended with other fibers, such as cotton, polyester and PA, making it difficult to effectively separate and recycle.^[^ ^145^, ^187^ ^]^ While elastane is not inherently unsuitable for recycling, the challenges associated with its composition, processing, and current recycling technologies make it less amenable to traditional recycling methods. Industry efforts are underway to develop more effective recycling solutions for spandex and similar synthetic fibers^[^ ^46^ ^]^, but only glycolysis processes are currently used on a large scale.^[^ ^124^, ^131^ ^]^ A brief comparison of the mechanical and chemical recycling of elastane is shown in **Table** **11**.

**Table 11:** Comparison of mechanical and chemical recycling of elastane fiber.

Sustainability aspect	Mechanical recycling	Chemical recycling
Environmental Impact	‐ Lower energy consumption ‐ Contamination issues ‐ Degradation of properties ‐ Lower waste generation	‐ Varies by process ‐ Can be lower energy ‐ Handles blended materials more effectively ‐ Potential for higher‐quality monomers ‐ Hazardous byproducts possible
Efficiency and yield	‐ Challenging due to elastic nature ‐ Low yield due to degradation ‐ Difficulty separating elastane from other materials	‐ Efficiency varies by process ‐ Can achieve higher yields ‐ Requires more complex processing and energy inputs
Scalability	‐ Well‐established ‐ Existing infrastructure can be adapted	‐ Still in early development phase ‐ Challenges in scaling up
Commercial viability	‐ Limited market demand ‐ Economically viable only for low‐value applications	‐ Growing market demand for high‐value applications ‐ Economically viable for high‐value applications

Today, bio‐based fibers have the potential to be more environmentally friendly to recycle than synthetic fibers. However, there are important nuances and challenges to consider.

For the production of bio‐based PAs, monomers derived from biomass, such as diamines and diacids, are considered as building blocks. For example, monomers such as AA and HMDA can be produced from renewable glucose feedstocks^[^ ^188^ ^]^ and via microbial fermentation.^[^ ^189^ ^]^ In the case of AA, most of the building blocks are produced from vegetable oils,^[^ ^190^ ^]^ with sugar conversion using yeasts. For example, cis, cis‐muconic acid, a polyunsaturated dicarboxylic acid, can be produced renewably by bioconversion of sugars and lignin‐derived aromatics using *Pseudomonas putida* strain KT2440. AA is produced catalytically from muconic acid.^[^ ^191^ ^]^ **Table** **12** describes some of the bio‐based PAs that are available on the commercial market.

**Table 12:** Some commercially available bio‐based PAs for textile industry.

Brand name	Producer	Bio‐based content	Key features	Application
TERRYL	Cathay Biotech	Made from renewable plant raw materials and has a bio‐based content of 45–100%	‐ Made from renewable plant materials ‐ Excellent spinning and finishing properties ‐ Easy to dye at low temperatures. ‐ Soft and skin‐friendly feel ‐ Good moisture and sweat absorption ‐ Good wear and weather resistance ‐ Natural flame‐retardant properties	Clothing, bags, carpets, work suits, and tents
Bio Amni	Solvay	Partially bio‐based polyamide 5.6 from sugar	‐ Produced using sugar to make monomers ‐ Reduces use of fossil fuels ‐ Similar sweat absorption to cotton ‐ Reduced water usage compared to traditional cotton ‐ Lower greenhouse gas emissions than standard polyamide	Sustainable textile
Q‐GEO	Fulgar	46% of the fiber is produced from non‐edible corn	‐ 50% more absorbent than standard polyamide ‐ Ultra fast drying ‐ Flame retardant properties without additives	Textiles requiring high performance and comfort
Bio based PA6.10	Nexis Fibers	63% of the polyamide comes from castor oil plant	‐ Reduced reliance on fossil fuel‐based materials	Clothing, sportswear, and technical textiles
Willskin Biobased	Balas textile	Made from castor oil. Bio‐based content of 100%	‐ Good breathability ‐ Fast drying ‐ Water repellent and chlorine resistant	Technical textiles
Ultramid PA	BASF	Derived from renewable resources (biomass).	‐ Made from renewable resources (biogas, bio‐naphtha) derived from organic waste or vegetable oils. ‐ Reduces the use of fossil fuels ‐ Lower greenhouse gas emissions than standard polyamide	Sustainable textile

While bio‐based elastane options are not yet as widely available as traditional elastane, they represent a significant step forward in creating more sustainable stretch fibers for the textile industry. As the demand for environmentally friendly materials increases, we can expect to see greater adoption and further development of these alternatives. Over the past decade, studies have been conducted to replace fossil‐based isocyanates with bio‐based molecules to obtain non‐isocyanate polyurethane (NIPU).^[^ ^192^, ^193^, ^194^, ^195^, ^196^, ^197^ ^]^ E. Pichon and co‐workers^[^ ^197^ ^]^ synthesized NIPUs by transcarbamoylation reaction. In this reaction, dicarbamate monomers were reacted with bio‐based polyether polyols (derived from bio‐based 1,3‐propanediol) and a tertiary amine as an internal dispersant, resulting in properties such as water resistance, durability, and low volatile organic compound (VOC) emissions, making them highly suitable for textile applications.

**Table** **13** provides a brief overview of current and emerging bio‐based elastane options in the textile industry, highlighting their key properties, bio‐based content and environmental benefits. As the industry continues to innovate, more options and improvements in these sustainable alternatives to traditional elastane can be expected.

**Table 13:** Some commercially available bio‐based elastane brands for the textile industry.

Brand name	Producer	Key features	Bio‐based content	Environmental benefits
Creora bio‐based	Hyosung TNC	Made from corn‐derived material Global production planned	Natural material derived from corn instead of coal	23% reduction in carbon emissions
				39% reduction in water use
				Eco Product Mark certified
LYCRA T400 EcoMade	The Lycra Company	Combines stretch with recycled and plant‐based materials	70% of the content derived from industrial cor	Uses recycled and plant‐based materials
Eco LYCRA T400	The Lycra Company	Maintains comfort and shape‐holding characteristics	≈68% (recycled and bio‐based)	Uses recycled plastics and renewable bio‐based resources
Original Bio‐based LYCRA	The Lycra Company	World's first bio‐based elastane (introduced in 2014)	70% derived from industrial corn	Derived from industrial corn
NeoLast™ Fibers	Celanese	Solvent‐free melt‐extrusion process	Not bio‐based, but eco‐friendly alternative	Eliminates hazardous chemicals in production
		Made from thermoplastic elastoester polymers		Potentially easier to recycle in blended fabrics
Future Bio‐based Elastane	Hyosung & Geno Partnership	Production planned for 2026	1,4‐butanediol (BDO) derived from sugar cane	Aims for fully integrated manufacturing from renewable raw material to fiber

In summary, while bio‐based fibers hold promise for more environmentally efficient recycling, their overall sustainability depends on several factors, including production methods, recycling infrastructure, and end‐of‐life management. Further research and development is needed to realize their full potential and to overcome existing challenges to large‐scale adoption and recycling.

## 5 Summary and Outlook

In the textile industry, PA and elastane are two of the most widely used synthetic fibers after polyester. Their global production is increasing every year due to their various applications. However, their accumulation poses environmental problems due to the difficulties in recycling these materials. Some challenges in recycling synthetic fibers are on the one hand, the degradation difficulties, such as the chemical structure and physical properties of PA and elastane make them not easy to degrade. On the other hand, the environmental impact, as the use of catalysts, chemical reagents and solvents in recycling processes can cause damage to marine and terrestrial ecosystems.

Recycling processes are designed to reuse and produce another material from waste. One of the most common types of recycling is thermo‐mechanical recycling, which involves the reuse and reshaping of synthetic fiber waste. Chemical recycling is a process that recovers monomers from synthetic polymers to synthesize second generation polymers. However, it can cause environmental damage. Biological recycling is an emerging field of research that uses microorganisms and enzymes to recycle polymers such as polyethylene terephthalate (PET), which is used in plastic bottles and polyester textile fibers.^[^ ^198^ ^]^

Enzymatic depolymerization of synthetic polymers is being studied to recover the monomers and reuse them in order to complete the recycling loop. The use of enzymatic engineering and other technologies can improve this type of recycling. Therefore, chemical recycling at laboratory scale shows monomer recovery at more than 90%, while biological recycling, due to the specificity of enzymes, still does not reach the efficiency of chemical recycling.

The introduction of biodegradable polymers in industry is a growing trend, but their production cost is still not viable to completely replace synthetic fossil‐based polymers. Bio‐polyamide (Bio‐Nylon) is currently being produced and industries are now producing elastane (Lycra) that is 50% biodegradable. While some bio‐based polymers are biodegradable, not all are.

Microbial degradation aims to break down materials into products that can be assimilated by biomass. While this topic is still under investigation, P. Buchholz and co‐workers^[^ ^199^ ^]^ have collected and integrated a list of microbial enzymes for plastics degradation into a comprehensive open‐access database. Thus, the appropriate approach to fiber recycling may involve a strategy that includes the improvement of recycling technologies (biological, chemical, and mechanical), the design of products to facilitate recycling and the use of sustainable materials and processing techniques. Recycling management can show great promise by combining methods to address the complex challenges of textile waste.

## Conflict of Interest

The authors declare no conflict of interest.
